# Notes on Myotomy and Tenotomy

**Published:** 1841-09-18

**Authors:** Reynell Coates

**Affiliations:** Philadelphia


### MEDICAL EXAMINER.

DEVOTED TO MEDICINE, SURGERY, AND THE COLLATERAL SCIENCES.

No. 38.] PHILADELPHIA, SATURDAY, SEPTEMBER 18, 1841. [Vol. IV.

### ORIGINAL COMMUNICATION.

### NOTES ON MYOTOMY AND TENOTOMY.

BY REYNELL COATES, M. D.

To the Editors of the Medical Examiner.

Gentlemen,—In my last I proposed to say a few words upon the subject of the objections urged against the mechanical treatment of club-foot without resort to tenotomy.

Were we to depend upon the American authorities who have been most strenuous in the advocacy of the knife, we might be led to conclude that cases of cure without such means were extremely rare, and that the few instances of success were almost exclusively confined to cases treated in early infancy. Passing by the obvious inferences to be drawn from the report of Dr. Brown, of Boston, let us examine some of the positions of our friend Professor Mütter. In treating of the form of club-foot which he evidently regards as the most manageable—that resulting from nearly simple extension, and an angular retraction of the anterior half of the foot, (pes equinus, in its first stage,) he says, when speaking of an apparatus for gradual flexion, successful in children under the year:—

"In children between the ages of 1 and 6, the same apparatus will answer in time, but I would not hesitate in such cases about the division of the tendon in fault." *Lecture on Loxarthans*, Philad. 1839, p. 58. Again, " When a person is advanced in life, and labours under this degree of pes equinus, he can only be relieved, *in a short time*, by a section of the tendo Achilles. Cases are reported in which the spring shoe of Scarpa, the sabot of Venel, and other contrivances have, with much suffering, and after a long time, brought down the heel; but do not, I beg of you, subject your patients to any such treatment; divide the tendon in all such cases."—*Ib.*

After impressing the same advice still more forcibly in the second stage, he remarks :

" In pes equinus of the third degree, (Talapes Equinus Verus of Mr. Little,) occurring at birth, I advise you to divide the tendo Achilles at once," &c. p. 63.

The principal objections to dependence on the apparatus alone, here appear to consist in the time occupied and the pain given. I shall comment upon these objections, with others, in the proper place—but it may be remarked with propriety here that all the weight of authority goes to show that congenital cases of this form of club-foot are very rare. It may occur at almost any period of life, and often commences after puberty, from obvious and well known causes inducing spasmodic contraction of the three extensors of the foot. There is a period in the history of this affection, *if resulting from spasm*, when mere mechanical extension might be altogether unwarrantable, and under certain circumstances tenotomy might be not only admissible but decidedly desirable. But this is a debateable question. I will endeavour to touch upon it hereafter. There is another period at which spasm no longer exists, and in which, at certain ages, the propriety of the employment of the knife before the attempt at cure by other means without its aid, depends upon the principles more commonly involved in all the forms of club-foot.

In relation to the saving of time effected by tenotomy, the value of this advantage depends entirely upon the question of the positive evils likely to result from the operation—evils which, *theoretically at least*, are considerable, even when the tendo Achilles is alone divided, and much greater when the fascia plantaris shares the same fate with the promptitude recommended by the sect of surgeons with whom Dr. Mütter expresses his accordance in opinion. Of this, also, more hereafter.

As regards the painfulness of the mechanical extension—always excepting the cases in which there exists spasmodic contraction of the muscles of the calf—it is necessary to consider in the comparison of methods, the relative merits of apparatus, and how far defects may be remedied in all that are before the public; nor is it improper to make some allowance for the want of mechanical aptitude, and sometimes the deficiency of physiological and therapeutic tact on the part of the practitioner from whose results conclusions have been drawn :—Not every operative surgeon, nor even every one of high and just pretensions, can apply a difficult complication of bandages, steel and leather, with discretion. But the necessity for mechanical force is not removed by the section of one or many tendons. The rigidity of the muscles is, in most cases, neither the only, *nor the principal obstacle* to the removal of the deformity. This subject will be discussed when I refer to the condition of the bones and ligaments interested in the malposition of the parts. But let me make some further reference on this especial point. Speaking of " the second degree of pes equinus," when the patient is somewhat advanced, Dr. Mütter remarks :

" In these cases, I have also found that the simple stretcher already described, did not possess sufficient *force* for the elevation of the toes; and besides this, it is necessary, from the great deformity of the foot, to employ a more complicated apparatus than a garter, to fix it upon a foot-board. It is, moreover, of great importance, in cases where it becomes necessary to employ *much force*, to have an apparatus so constructed that it may be *regularly* and *gradually* kept up, and increased and diminished at will," &c. p. 61.

In at least one of the cases mentioned by M. Bouvier, in *Mem. de l'Acad. Roy. de Medicine*, 1838—and in Delpech's case narrated in *Clin. Chir.*, and completed from observations made after many years, by M. Bouvier, in the same paper, and in *Dict. de Med. Prat.* art. *Pied-bot*, the mechanical means necessary after the division of the tendo Achilles were productive of severe suffering.

Hear also Dr. Detmold, of New York, whose large experience—notwithstanding Dr. Chadbourne's idea that the profession had settled down generally into a belief of the necessity of tenotomy—is any thing but an exclusive advocate of the knife. Speaking of extension after the resection and commencing reunion of the tendo Achilles, he remarks:

"We say, a moderate degree of extension is alone indicated; for if it exceed those limits which are dictated by prudence, and by the consideration that we are treating a living subject, and a contractile muscle that will yield to a certain point,—but on this point being exceeded, will react against the extension,—and that if this is persisted in, the whole system will sympathise with the part, *and convulsions and other serious nervous symptoms will be the consequence.*"—*New York Jour. of Med. and Surg.*, 1840.

It is evident, then, that the division of tendons does not remove the necessity for mechanical extension requiring care and circumspection, and that accidents of a character similar to those objected against the use of extending apparatus alone, do actually occur, at least occasionally, when the combined plan of treatment is resorted to.

The question of pain, therefore, in the comparison between these contending methods, resolves itself in this: Which of the two modes of treatment is productive of most suffering to the patients? I have enjoyed some opportunities of investigating this matter by examining a few patients who have been treated by some of the best operators in each, and this experience, added to certain boyish recollections alluded to in my last communication, convince me that it is possible, in very many instances, to effect what is usually considered as a cure of club-foot without resort to tenotomy, and without inflicting *so much pain* as has often followed the latter process. This has been, however, in part the result of the greater simplicity of the apparatus employed, and, in part, the absence of a desire for too speedy a termination of the labour. Cæteris paribus, there is certainly nothing in *the immediate consequences* of tendon cutting which should enhance the pain of treatment, except as practised by a few, whom it is merciful to spare, and who carve away at tendons, ligaments and fascia by the half dozen at a time, reporting their cases without even taking the trouble to enumerate the divided parts. I will go further, and acknowledge that, in every instance, the division of these bonds must diminish the resistance to be overcome, and lessen the amount of force required *in certain stages of the treatment.* But will this slight advantage repay the subsequent evils? We shall see. In passing, let me here remark, that all the force which is warrantable in any case, when properly applied, does not exceed the endurance of the patient or the parts impressed.

In valgus "of the second and third degrees," Dr. Mütter directs the division of the tendon in every case occurring or continuing beyond the age of six years. "It will be useless to attempt the cure by mechanical means." p. 56.

Waiving the remarks on the milder cases of varus, he remarks of the third stage or "degree" of this affection:

"Such is the contraction of the tendo Achilles, tibial tendons, and the fascia plantaris, that even *at birth*, we find mechanical measures *alone*, almost invariably failing to do any good." p. 50.

I shall not pause to seek authorities upon this question,—one on which I know that many highly eminent men agree with him most fully,—but simply remembering that the complete inversion of the fore part of the foot at birth must be extremely rare, it is proper to observe that the commonly received opinion of the incurability of complete varus after puberty, is exceedingly unlikely to bear the test of future experience, however it may have been in former years. Assuredly there are cases of this affection now walking the streets of Philadelphia who have undergone *what is called a cure* of this stage of varus, with undivided tendons, at an age many years removed from birth. I have treated and have met with several in early life, and some have recently been presented to me in which the deformity had passed the limits prescribed for varus of the third degree, and the age approximated to puberty.

It may be asked, why I use the phrase "what is called a cure."—Simply for this reason. I have never seen a case treated *with or without the aid of tenotomy*, in which the tarsal bones resumed completely their proper relative position, or one in which the ankle or the instep had acquired perfect freedom of motion, except in children cured when under three years old, and in whom the symptoms of deformity had disappeared in after life. Nor can I, in the face of all the vices of nutrition, and changes of the articulating surfaces which invariably occur in cases of a few years standing, believe in such results as are represented as occurring at the end of a few days or months. There is a most unguarded use of the word *cure* observable among surgeons generally, and especially among those who publish orthopœdic observations. It is exceedingly dangerous sometimes to prove too much! How often do we see the pompous certificates of respectable and worthy, though ignorant men, in favour of empirics who are continually accomplishing *the impossible*,—the natural bone-setters who restore a bone to a cavity which for years has ceased to exist, and other equally miraculous performances. Can any man expect a well-informed anatomist and physiologist to credit the assertion that, after the proper head of the astralagus has lost one half its cartilage; after the scaphoid bone has formed a new and regular joint with the maleolus; when the body of the astralagus has become a wedge-shaped mass and the tibia is articulated with the calcis,—can he be called upon to believe that, after a dozen new ligaments, all short and dense, are formed between points which should be far asunder, these bones, joints, and ligaments are all restored to their proper position and relations in a few days, by applying straps, buckles, and magical little pieces of steel, either with or without division of the tendons of the triple extensor, the tibialis anticus, the flexor communis, or the fascia plantaris, or all of them? Yet, let me say to the anonymous correspondent of the Boston Journal, who seems to place great confidence in the weight of authority, that I could bring him high,—ay! even among the highest authority, both European and American, for such most modest pretensions to success. In surgery, and indeed, in every thing but law, one fact,—*if it be a fact*—will outweigh the authority of all mankind—academies and all. And sometimes a few grains of common sense, and a cool recollection of the laws of life, will render the mere examination of pretended facts unnecessary, though they may be backed by the boldest and the greatest. Did not Cuvier report in favour of Barry's absurd theory of the pulse, directly in the teeth of twenty thousand facts of his own discovery, because, forgetting other *well ascertained facts*, he thought there was *plausibility* in certain *assumed facts* of the English theorist, *which contravened the unalterable laws of statics?*

Delpech's patient (it was a case of pes equinus, with division of the tendo Achilles) walked rapidly and firmly, without fatigue, *and hopped vpon the affected foot*, yet it would not admit of flexion beyond the right angle, *twenty years after the operation.* The exaggeration of the tarsal arch remained unchanged from its condition at the end of three years. There was still an "*outward* inclination of the foot" produced by the course of treatment, and uncontrollable by lateral splints; and the patient wore wool in his shoe to relieve the undue pressure on the head of the first metatarsal bone. (*Mem. de l' Acad.*, tome 7.) In many of Bouvier's cases the remaining lateral arcuation and the high instep after the treatment of valgus by tenotomy, are carefully noticed. I have seen no case in which they were absent after either mode of treatment. Yet many writers tell us that after operation with the knife, and sometimes even after simple mechanical extension in advanced cases, their patients "walk very well;" have the affected foot "as perfectly formed" as its fellow; "are perfectly cured," &c. in from thirty days to six months of time! If any man believe that the judgment, in these instances, has not been warped by the anxiety for success, display, or notoriety, his faith in the triumphs of art over nature exceeds the limits of safe surgical principle!

It is one of the unfortunate circumstances in the endeavour to investigate the rival claims of the two methods of treatment under review, that our personal evidence of the innoxiousness of the operation of tenotomy and its consequences, together with the degree of pain resulting from the after treatment, must be derived mainly from such authorities as have been guilty of this justly censureable carelessness in their reports. It is often safer to fall back upon the well known laws of vital action, analogy, and unprejudiced reason, than to trust to *half-observed and less than half-reported cases*, where the patients walk or limp away from observation before the real issue of the case can possible be known. Yet such is the nature of much of the evidence on which rests the growing popularity of tendon and muscle cutting, not only in club-foot, but in deformities of spine, false anchyloses, stammering, and strabismus. I might extend the catalogue, but "*sufficient unto the day is the evil thereof.*"

But this long letter threatens to prove too severe a tax upon your space and patience. This subject grows upon me, and it will be best to defer for another number the necessary remarks on the real nature of the forces to be overcome in the treatment of club-foot, a subject which must be properly discussed before we can reason otherwise than empirically on the advantages of tenotomy.

An apology is due to Dr. Mütter for the selection of his lecture as the text of these strictures, which certainly can have no mere individual application. His little volume and that of Dr. Detmold, are the most convenient in point of size for frequent reference, and as his opinions are those of a class which numbers many names of very high repute, I have taken his article as the expression of the views of Stromeyer, Dieffenbach, Guérin, Little, Coates, (of London,) Mott, &c. With this assurance I must close for the present, or delay the press.

*Philadelphia*, September, 1841.

